# 54-Year-Old Female with a Syncopal Episode

**DOI:** 10.5811/cpcem.2018.1.37086

**Published:** 2018-01-29

**Authors:** Nicole Cimino-Fiallos, Wan-Tsu W. Chang, Laura J. Bontempo, Zachary D.W. Dezman

**Affiliations:** * University of Maryland Medical Center, Baltimore, Maryland; † University of Maryland School of Medicine, Department of Medicine, Baltimore, Maryland

## Case Presentation (Dr. X)

A 54-year-old woman presented to the emergency department (ED) with a complaint of syncope. The patient was unable to offer any history, so all information was obtained from her friend, paramedics, and past records. Her friend, who accompanied the patient to the ED, reported that the patient had been “feeling unwell,” vomited twice, and then went to bed earlier that day. A few hours later, the friend heard a loud thud and subsequently found the patient unresponsive on the floor. When paramedics arrived at the scene, the patient was unresponsive. They gave her 50 gm of dextrose intravenously because her capillary blood glucose was 17 mg/dL. She regained consciousness, but continued to be altered during her transport to the ED.

Her past medical and surgical histories included hypothyroidism, anemia and a Cesarean section 17-years prior, complicated by postpartum hemorrhage. Her friend was adamant that the patient has no history of diabetes mellitus (DM). Her only known medication was levothyroxine (dose unknown). She had no known drug allergies and her last known menstrual period was before the birth of her son.

The patient works as a custodian and lives with her son, a recent high school graduate. The patient’s friend stated that the patient does not smoke, drink alcohol, or use recreational drugs. The friend stated that the patient had not had any recent injuries, headaches, illnesses, or sick contacts. A complete review of systems could not be obtained due to the patient’s altered mental status.

Physical examination revealed a well-developed, thin woman resting on a stretcher. She had a temperature of 98.5° Fahrenheit with a heart rate of 60 beats per minute, and a blood pressure of 146/82 mmHg. She was breathing 16 breaths per minute with an oxygen saturation of 100% on room air. Her body mass index was 18.5kg per meter squared. Examination of the head, eyes, ears, nose and throat showed that the patient was normocephalic and atraumatic, without evidence of intraoral or external trauma. Pupils were three mm, equal, round and reactive to light. Lungs were clear to auscultation with equal breath sounds bilaterally. Cardiac exam revealed a regular rate and rhythm, without murmurs, rubs, or gallops. The patient’s abdomen was soft with normal bowel sounds and without distention, tenderness, rebound, guarding, or organomegaly. The extremities did not have any edema or evidence of trauma. There were 2+ pulses throughout all the extremities and all were warm, well perfused and without evidence of tenderness to palpation. Neurological exam revealed an awake patient oriented only to self and able to follow simple commands. The patient was unable to participate in detailed cranial nerve testing. Genital exam showed normal female genitals with pubic alopecia. Her skin was warm and dry. Cranial hair was thin.

**Image 1:**
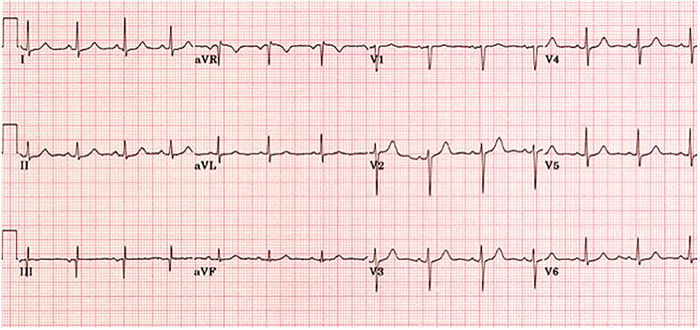
Patient’s initial electrocardiogram showing a normal sinus rhythym. Obtained while in the emergency department.

**Image 2:**
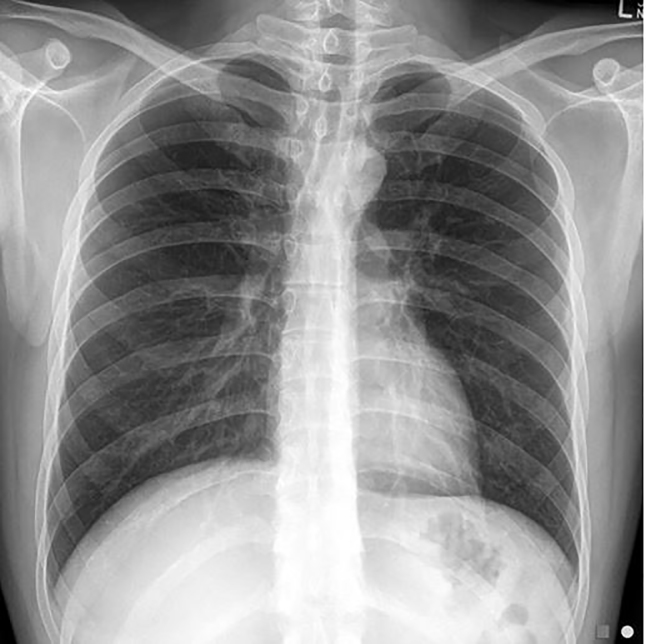
Radiograph of the patient’s chest while in the emergency department showing no acute disease (posterior-anterior view).

**Image 3:**
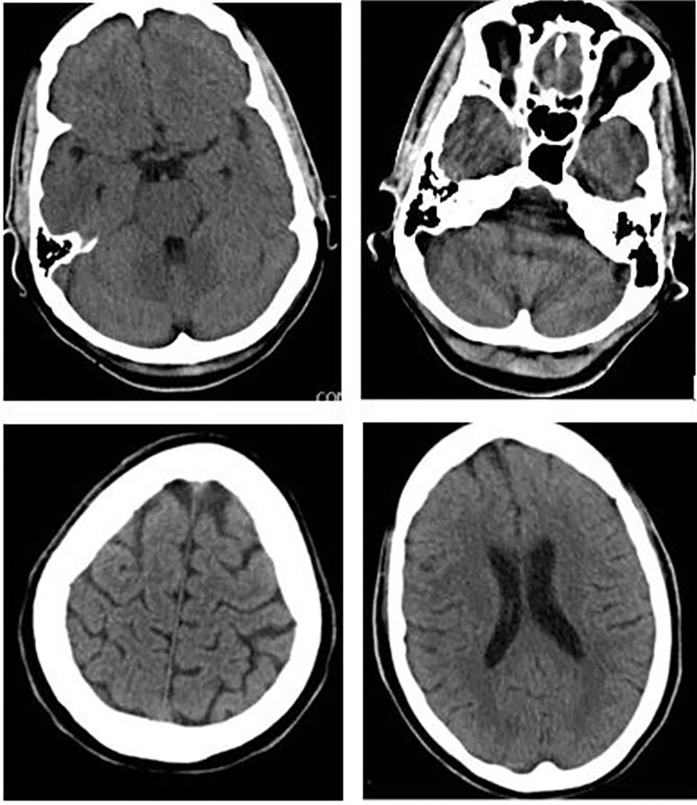
Representative images from the patient’s computed tomography of the head, showing no acute disease.

Laboratory results are shown in table. Her electrocardiogram (ECG) is shown in Image 1. A chest radiograph (Image 2) and computed tomography (CT) of her head were obtained. Representative images of the CT are shown in Image 3.

Approximately an hour after arrival to the ED, the patient became unconscious and could not be aroused. Capillary blood glucose was 32 mg/dL. The patient was treated with dextrose 50g intravenously and regained consciousness.

A diagnostic test was then done, which confirmed the patient’s diagnosis.

## Case Discussion (Dr. Y)

This is a 54-year-old female who was found unresponsive and hypoglycemic with altered mental status. Patient has a past medical history significant for hypothyroidism, anemia, and prior Cesarean-section with hemorrhage. She does not have a known history of DM thus the cause of her presenting hypoglycemia is unclear at this time. On exam, the patient is alert, able to follow commands, though oriented to person only. She has dry, thin hair consistent with her known history of hypothyroidism.

In reviewing this case, one thing that stood out is the patient’s presenting hypoglycemia without a known diagnosis of DM. The patient did not present with any neurogenic symptoms of hypoglycemia such as sweating, shakiness, tachycardia, anxiety, or sensation of hunger. However, she did present with neuroglycopenic symptoms of hypoglycemia such as confusion and unresponsiveness.

The differential diagnosis of hypoglycemia can be categorized into exogenous drugs, increased glucose utilization, decreased glucose delivery, decreased glucose production, increased insulin production, and decreased insulin clearance. In patients with a history of DM, common causes of hypoglycemia are exogenous drugs such as sulfonylureas and insulin, and decreased glucose delivery such as with fasting. It is important to remember that sepsis can increase glucose utilization while renal failure can decrease insulin clearance, both leading to hypoglycemia. Thus, one should consider the full differential diagnosis of hypoglycemia even when dealing with a patient with known DM.

In patients without a history of DM, the etiology of hypoglycemia is more complex. Exogenous drugs including alcohol, beta-blockers, valproic acid, and salicylates can all cause hypoglycemia. Malaria can increase glucose utilization. One needs to consider decreased glucose production in patients with liver disease, Addison’s disease, or pituitary insufficiency. There may be increased production of insulin from insulinomas or islet cell hyperplasia. And finally, we also have to be sure that we are not dealing with pseudohypoglycemia in the setting of leukocytosis, thrombocytosis, or erythrocytosis.

This patient’s history does not suggest any exogenous drugs as the cause of her hypoglycemia, though we cannot be certain that she does not take salicylates as an over-the-counter analgesic. Her clinical presentation does not suggest sepsis or malaria. She also does not have any stigmata of liver disease on her physical exam. At this time, we are still left with a wide differential.

Her laboratory studies are notable for an anemia consistent with her history. However, she has a low thyroid stimulating hormone (TSH) and free thyroxine level, which is not suggestive of primary hypothyroidism. She also has a hyponatremia of unclear etiology. The remainder of her diagnostic studies including ECG, chest radiograph, and noncontrast head CT are unremarkable.

When we summarize the facts, we have a 54-year-old female with no history of DM, who has persistent hypoglycemia associated with hyponatremia. While she has a history of hypothyroidism her thyroid function studies are not consistent with a primary hypothyroidism, but rather, secondary or central hypothyroidism. When we review the case for any additional clues, we find that her last menstrual period was in 1996, approximately 19 years ago, similar to the age of her son. She has not had a total hysterectomy since the birth of her son, though she did have a Cesarean-section with hemorrhage. Could she have had a postpartum hemorrhage with pituitary infarction leading to amenorrhea?

Supporting evidence for diagnosis of hypopituitarism are the patient’s central hypothyroidism, amenorrhea due to inadequate gonadotropin production, and secondary adrenal insufficiency due to inadequate adrenocorticotropic hormone production. It is important to note that the patient does not have any hypotension or hyperkalemia, thus suggesting that she has intact mineralocorticoid function. Her glucocorticoid function, however, is affected as evidenced by her hypoglycemia. Her hyponatremia is caused by an inappropriate secretion of antidiuretic hormone due to the lack of negative feedback from cortisol.

The study of choice to make the diagnosis of hypopituitarism relating to pituitary infarction would be magnetic resonance imaging (MRI) of the brain to demonstrate an empty sella. If the patient were to have developed symptoms of hypopituitarism early after her postpartum hemorrhage, an MRI can demonstrate an enlarged pituitary with peripheral enhancement. Work-up of her endocrinopathies are important to evaluate the extent of her hypopituitarism and to direct treatment.

## Case Outcome (Dr. X)

**Image 4:**
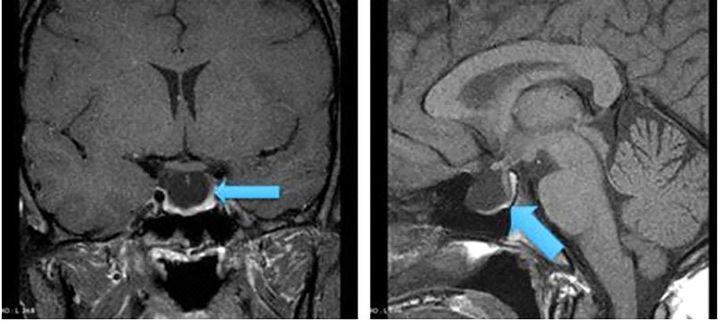
Representative images from the patient’s computed tomography of the head, showing no acute disease.

A brain MRI was performed, which revealed an empty sella turcica (Image 4). The patient was diagnosed with Sheehan’s syndrome (postpartum hypopituitarism) and was admitted to the hospital for further evaluation and treatment. She was treated with steroids due to her adrenal insufficiency and her levothyroxine dosage was adjusted. She was discharged from the hospital with endocrinology follow-up and was back at work within two weeks.

After a massive postpartum hemorrhage, the pituitary gland can infarct resulting in complete or partial hypopituitarism and subsequent endocrinopathies. The size of the infarction directly correlates to the extent of the syndrome, which was identified by Dr. Harold Sheehan in 1937. There are several theories as to why the syndrome occurs.^1^ The pituitary grows in response to constant high levels of estrogen during pregnancy (lactotroph hyperplasia). Because the pituitary resides in an enclosed space, this growth causes increased intrasellar pressure. The location of the pituitary gland’s blood supply also makes the gland vulnerable to ischemia. Another possible explanation is that the gland undergoes a massive thrombosis from either the hypercoagulability of pregnancy or arterial vasospasm from bleeding during delivery.^2^ Some patients may form anti-pituitary autoantibodies.^3^

## Epidemiology

This syndrome mostly occurs in developing countries. Patients in those countries have less access to obstetrical care and are more likely to have massive hemorrhage during delivery.^2^ Similarly, the group of patients in the United States who most often have Sheehan’s syndrome are those who deliver outside of a medical setting. As a part of their prenatal care, obstetricians are able to anticipate which patients are at high risk for bleeding based on placental location, past history, coagulation status, etc. Obstetricians can also help to limit the amount of bleeding that occurs during delivery while aggressively transfusing the patient when bleeding occurs.^4^

## Presentation

The most common presenting symptom of Sheehan’s syndrome is the failure to menstruate or lactate after delivery. Other symptoms of this syndrome include axillary and pubic hair loss, fatigue, cold intolerance, weakness, breast atrophy, decreased libido, adrenal insufficiency, diabetes insipidus and possibly osteoporosis.^5^ A patient’s symptoms can vary due to the extent of pituitary necrosis and the specific areas of the pituitary that are necrosed^6^,^7^. There are case reports of patients not being diagnosed until several decades after the birth of their last child^6^,^7^. Many women can be asymptomatic until a stressor precipitates an endocrinological crisis such as myxedema coma or adrenal insufficiency. The continued production of aldosterone by the adrenal gland can also mask some symptoms of Sheehan’s syndrome. Other causes of hypopituitarism include pituitary adenomas, autoimmune or lymphocytic hypophysitis, and congenital causes. The patient in this case had several clues to suggest the diagnosis: her last menstrual period was before the birth of her son, she had a history of hypothyroidism and postpartum hemorrhage, and her exam was suggestive of hypothyroidism. Her testing revealed a low TSH level, hypoglycemia and hyponatremia in the presence of normokalemia, suggesting a glucocorticoid deficiency without a disturbance in the mineralocorticoid pathway.

Adrenal insufficiency is a common presentation of Sheehan’s syndrome. The patient will present with recurrent hypoglycemia.^8^,^9^ As the need for energy increases, the adrenals will not release cortisol as in a normal person. Without cortisol, the liver will not convert glycogen to glucose and the patient will present with hypoglycemia. In addition to hypoglycemia, the patient can also present with hypotension, orthostasis, fatigue, hypopigmentation and sometimes sudden death. The differential diagnosis for causes of adrenal insufficiency should include Addison’s disease, pituitary adenoma, Sheehan’s syndrome, and infection.

## Diagnostic Testing

The patient’s thyroid function tests, including serum triiodothyronine, free thyroxine, TSH and cortisol should be checked in the ED. The patient’s endocrinologist may choose to test the patient’s cortisol, adrenocortioctropic hormone, follicle stimulating hormone, luteinizing hormone, estradiol and prolactin levels. MRI of the brain is the definitive test. In early disease, the MRI will show an enlarged non-hemorrhagic pituitary gland. In chronic disease, the MRI will show an empty sella turcica, due to gland necrosis and atrophy.

## Treatment

The presentation of Sheehan’s syndrome is a result of the constellation of endocrinopathies that occur when the pituitary gland is non-functional. The treatment for this disease is to replace the missing hormones. If the patient has adrenal insufficiency, the patient will need steroids, specifically hydrocortisone. There is no need to replace mineralocorticoids as the renin-angiotensin system should be unaffected. If the patient is having recurrent hypoglycemia, she should be started on dextrose containing intravenous fluids. If the patient is awake enough, she should eat a meal of complex carbohydrates. If the patient presents to the hospital in myxedema coma, she should first be given glucocorticoids to prevent an adrenal crisis and then be treated with levothyroxine.^2^ The patient’s primary care doctor may choose to treat with estrogen to prevent osteoporosis. Growth hormone replacement is very expensive and has not been shown to improve outcomes.

## Final Diagnosis

Sheehan’s syndrome resulting in adrenal crisis.

## Take-home Points

- Recurrent hypoglycemic episodes, especially in the context of hyponatremia, should prompt the clinician to consider adrenal insufficiency.
- Immigrants from countries without access to obstetrical care may present with symptoms of Sheehan’s syndrome.
- Patients with Sheehan’s syndrome can present with emergencies such as adrenal crisis or myxedema coma.
- MRI is the diagnostic test of choice for Sheehan’s syndrome and the images in early disease will look different from late disease.
- While Sheehan’s syndrome has no cure, the goal is to treat the resulting endocrinopathies.

## Figures and Tables

**Table 1:** Initial laboratory results of patient presenting with syncope.

Complete blood cell count	Values
White blood cell count	13.1 K/mcl
Hemoglobin	9.7 g/dL
Hematocrit	29.8%
Platelets	196 K/mcl
Complete metabolic panel	
Sodium	128 mmol/L
Potassium	3.8 mmol/L
Chloride	104 mmol/L
Bicarbonate	28 mmol/L
Blood urea nitrogen	14 mg/dL
Creatinine	0.9 mg/dL
Glucose	122 mg/dL
Alanine aminotransferase	32 u/L
Aspartate aminotransferase	8 u/L
Alkaline phosphatase	80 u/L
Total bilirubin	0.7 mg/dL
Total protein	7.6 g/dL
Albumin	4.1 g/dL
Calcium	8.9 mg/dL
Additional laboratory tests	
Free thyroxine	1.18 ng/dL
Thyroid stimulating hormone	0.054 mIU/L
Troponin	<0.02 ng/mL
Urinalysis	
Color	Straw
Ketones	1+
Nitrite	Negative
Glucose	1+
Protein	Negative
Blood	Negative
Leukocyte esterase	Negative
Human chorionic gonadotropin	Negative
Urine drug screen	Negative
Acetaminophen level	<10 mcg/mL
Salicylate level	<3 mg/dL
